# Current Limitations and Candidate Potential of 5-HT7 Receptor Antagonism in Psychiatric Pharmacotherapy

**DOI:** 10.3389/fpsyt.2021.623684

**Published:** 2021-02-18

**Authors:** Ruri Okubo, Toshiki Hasegawa, Kouji Fukuyama, Takashi Shiroyama, Motohiro Okada

**Affiliations:** Division of Neuroscience, Laboratory Department of Neuropsychiatry, Graduate School of Medicine, Mie University, Tsu, Japan

**Keywords:** antipsychotics, bipolar disorder (BD), cognition, lurasidone, schizophrenia, 5-HT7, transmission

## Abstract

Several mood-stabilizing atypical antipsychotics and antidepressants weakly block serotonin (5-HT) receptor type-7 (5-HT7R); however, the contributions of 5-HT7R antagonism to clinical efficacy and pathophysiology are yet to be clarified. A novel mood-stabilizing antipsychotic agent, lurasidone exhibits predominant binding affinity to 5-HT7R when compared with other monoamine receptors. To date, we have failed to discover the superior clinical efficacy of lurasidone on schizophrenia, mood, or anxiety disorders when compared with conventional mood-stabilizing atypical antipsychotics; however, numerous preclinical findings have indicated the possible potential of 5-HT7R antagonism against several neuropsychiatric disorders, as well as the generation of novel therapeutic options that could not be expected with conventional atypical antipsychotics. Traditional experimental techniques, electrophysiology, and microdialysis have demonstrated that the effects of 5-HT receptor type-1A (5-HT1AR) and 5-HT7R on neurotransmission are in contrast, but the effect of 5-HT1AR is more predominant than that of 5-HT7R, resulting in an insufficient understanding of the 5-HT7R function in the field of psychopharmacology. Accumulating knowledge regarding the pharmacodynamic profiles of 5-HT7R suggests that 5-HT7R is one of the key players in the establishment and remodeling of neural development and cytoarchitecture during the early developmental stage to the mature brain, and dysfunction or modulation of 5-HT7R is linked to the pathogenesis/pathophysiology of neuropsychiatric and neurodevelopmental disorders. In this review, to explore candidate novel applications for the treatment of several neuropsychiatric disorders, including mood disorders, schizophrenia, and other cognitive disturbance disorders, we discuss perspectives of psychopharmacology regarding the effects of 5-HT7R antagonism on transmission and intracellular signaling systems, based on preclinical findings.

## Introduction

In the body, 95% of tryptophan is metabolized by the kynurenine pathway and 5% by the serotonin (5-HT) pathway (1–4). Only 5% of 5-HT is distributed in the central nervous system (CNS), and the remainder is synthesized and degraded in peripheral tissues (3). In the CNS, serotonergic transmission plays a fundamental role in the pathophysiology of mood disorders and schizophrenia (5, 6). 5-HT receptor type 7 (5-HT7R) is one of the most recently (1993) identified members of the 5-HT receptor family (7–10) and is highly expressed in functionally relevant regions of the brain (11, 12). In the mammalian CNS, 5-HT7R is most predominantly expressed in the thalamus, and in the hypothalamus, hippocampus, prefrontal cortex, striatal complex, amygdala, and dorsal raphe nucleus (DRN) (13–19). Over the last two decades, preclinical studies have accumulated various findings highlighting that 5-HT7R is one of the key players in the regulation of mood, memory processing, cognition, and emotional perception, as demonstrated by various experiments using selective 5-HT7R antagonist and 5-HT7R knockout mice model (20–25). Moreover, the predominant expression of 5-HT7R in the limbic regions supports the hypothesis that 5-HT7R contributes to the regulation of memory processing, cognition, and emotional perception in association with several types of cognitive domains (14, 16, 18, 19, 25).

It has been postulated that 5-HT7R antagonism probably plays an important role in the clinical efficacy of several mood-stabilizing antipsychotics, as aripiprazole, clozapine, quetiapine, risperidone, and zotepine are known to antagonize 5-HT7R (Table 1) (18, 26, 27, 29–34, 37–39). Additionally, a novel antidepressant, vortioxetine, which is categorized as a 5-HT partial agonist reuptake inhibitor (SPARI), inhibits 5-HT7R (Table 1) (36, 39). However, the clinical potential of 5-HT7R antagonism is yet to be comprehensively elucidated as binding affinities of these conventional antipsychotics and vortioxetine are more highly sensitive to other 5-HT receptor subtypes than to 5-HT7R (Table 1). In contrast, a novel mood-stabilizing antipsychotic agent, lurasidone, is the only antipsychotic agent with the highest binding affinity to 5-HT7R when compared with other monoamine receptors (26) (Table 1). Therefore, to clarify crucial clinical targets of 5-HT7R antagonism for the treatment of neuropsychiatric disorders, including schizophrenia, mood disorders, and other cognitive disturbance disorders, in this review, we discuss the psychopharmacological perspectives of 5-HT7R antagonism, based on the preclinical findings of 5-HT7R antagonism and clinical evaluation of lurasidone to date.

**Table 1:** Receptor binding profiles of antipsychotic agents.

**Receptor**	**LUR**	**APZ**	**CLZ**	**PMZ**	**QTP**	**RIS**	**ZTP**	**BNS**	**VTX**
5-HT1AR	6.8	5.6	124	650	432	423	471	804	15.0
5-HT2AR	2.0	8.7	5.4	48.4	100	0.2	2.7	0.8	
5-HT3R	>1,000	630	241	>1,000	>1,000	>1,000	472	>1,000	3.7
5-HT7R	0.5	10.3	18.0	0.5	307	6.6	12.0	183	19.0
α2A	>1,000	74.3	37.0	>1,000	>1,000	16.5	180	(530)	
α2B	>1,000	102	26.5	821	90.0	108	5.4		
α2C	10.8	37.9	6.0	377	28.7	1.3	106		
D1R	262	>1,000	266	>1,000	712	244	71.0	>1,000	
D2R	1.7	3.3	157	0.3	245	3.6	25.0	0.1	
References	(26)	(27, 28)	(29, 30)	(31)	(32)	(28, 33)	(34)	(35)	(36)

*lurasidone (LUR), aripiprazole (APZ), clozapine (CLZ), pimozide (PMZ), risperidone (RIS), zotepine (ZTP), and blonanserin (BNS), and antidepressant vortioxetine (VTX) against serotonin (5-HT) type 1A (5-HT1AR), type 2A (5-HT2AR), type 3 (5-HT3R), and type 7 (5-HT7R) receptor, and dopamine receptors type 1 (D1R) and 2 (D2R). Data are equilibrium constant (Ki) values (nM)*.

## Clinical Evaluation of Lurasidone and Preclinical Potentials of 5-HT7R Antagonism

Lurasidone has been approved for the treatment of schizophrenia by the United States Food and Drug Administration (FDA), the European Medicines Agency (EMA), and Japanese Pharmaceuticals and Medical Devices (PMDA) (40). Additionally, lurasidone is has been approved by the FDA and PMDA, but not by the EMA, for the treatment of bipolar depression in monotherapy and combined with lithium or valproate in adults, and as monotherapy in children and adolescents (41).

### Schizophrenia

A network meta-analysis of placebo-controlled and head-to-head randomized controlled trials indicated that lurasidone significantly improves positive, negative, and depressive symptoms, and improves the quality of life and social functioning when compared with placebo (42). Therefore, the general efficacy of lurasidone for the treatment of schizophrenia seems to be comparable to other atypical antipsychotics; however, the crucial superiority and specific targets responsible for the clinical efficacy of 5-HT7R antagonism remain to be detected.

Moreover, the optimal dose outcomes of lurasidone remain unknown as this drug is being assessed further in ongoing clinical trials. Indeed, a dose-response meta-analysis demonstrated that the 95% effective dose (ED95) of lurasidone for acute schizophrenia symptoms was achieved at 147 mg/day, which was calculated by six dose-finding studies. However, the dose-response curve suggested that higher doses could be more efficacious than the highest dose tested so far (160 mg/day) (43) Therefore, in combination with these findings, the clinical evaluation of a higher dose of lurasidone is possibly more efficacious than that of the conventional rating scale.

It has been well-established that dopamine D2 receptor (D2R) antagonism with 5-HT receptor type 2A (5-HT2AR) antagonism or 5-HT1AR partial agonism, with well-known receptor binding profiles in atypical antipsychotics, contribute to the clinical efficacy of antipsychotics against positive and negative symptoms of schizophrenia (44, 45). Although each antipsychotic has subtly distinct receptor binding profiles, lurasidone reportedly exhibits a receptor binding profile like an atypical antipsychotic agent, because lurasidone is a potent partial agonist of 5-HT1A, as well as a D2R and 5-HT2AR antagonist (44, 45). A positron emission tomography (PET) study demonstrated that clozapine, olanzapine, risperidone, and ziprasidone display high 5-HT2AR occupancy even at a low dose (~70–80%), but dose-dependently increase D2R occupancy (46–48), whereas lurasidone preferentially displayed D2R, rather than 5-HT2AR, occupancy (48, 49). D2R and 5-HT2AR occupancy levels of lurasidone (80 mg) were ~70–80% and lower than 40%, respectively (48, 50). Consistent with the *in vitro* receptor binding profile of lurasidone to 5-HT1AR (6.8 nM), 5-HT2AR (2.0 nM), 5-HT7R (0.5 nM), and D2R (1.7 nM) (Table 1) (26), the approved dosage of lurasidone probably displays predominant binding to D2R and 5-HT7R over binding to 5-HT1AR and 5-HT2AR. Therefore, the clinical efficacy of relatively low doses of lurasidone can be evaluated as 5-HT7R antagonism, whereas the clinical effects of a relatively high dose of lurasidone are probably affected by additional effects associated with 5-HT2A antagonism, along with 5-HT1AR partial agonism.

Several clinical studies have reported that clinical targets of 5-HT7R antagonism might differ from those of conventional atypical antipsychotics (51–59). Notably, 5-HT7R variants are not associated with response to atypical antipsychotics in schizophrenia (60, 61). Furthermore, a recent meta-analysis study revealed a significant association between responses to positive and negative symptoms with lurasidone and functional polymorphism of 5-HT receptor type 1A (5-HT1AR), but not those of 5-HT7R (62). The candidate superiorities of lurasidone are an improvement of atypical antipsychotic-resistant cognitive impairments (55, 57, 63) and prevention of relapse/recurrence, resulting in an improvement in the quality of life (51, 52, 56, 58, 59, 64–66).

For schizophrenia, improvements in cognitive impairment by atypical antipsychotics are limited (67). It is well-known that the executive function cognitive domain is a critical antipsychotic-resistant cognitive domain (68). Both atypical antipsychotics, clozapine and olanzapine, slightly improved the executive function in schizophrenia (67, 69), but only lurasidone has been confirmed to improve executive function in patients with atypical antipsychotics-resistant schizophrenia (57). The overall effectiveness of lurasidone against treatment-resistant schizophrenia is reportedly considered to be almost equal to that observed with clozapine, olanzapine, and risperidone. However, a recent clinical study demonstrated that lurasidone improved several cognitive domains, including executive function in patients with atypical antipsychotics-resistant schizophrenia (especially clozapine-resistant schizophrenia) (57). Interestingly, the approved dose of lurasidone (80 mg/day) improved the executive functions in atypical antipsychotic-resistant schizophrenia rather than higher doses (57). In particular, the improvement of executive function by lurasidone was independent of improvements in the positive and negative syndrome scales (57). The improvement of executive functions (atypical antipsychotic-resistant cognitive domains) in atypical antipsychotic-resistant schizophrenia suggested that 5-HT7R antagonism plays an important role in the cognitive promoting effects of lurasidone during atypical antipsychotic-resistant cognitive impairment, rather than 5-HT2A antagonism or 5-HT1AR partial agonism. Therefore, the discrepancy between the therapeutic dose ranges of lurasidone for cognitive promoting action (55, 57, 63) and acute schizophrenia symptoms (43) suggests that relatively low doses contribute to the cognitive promoting effects via predominantly 5-HT7R antagonism, but the improvement of acute schizophrenia symptoms requires a relatively high dose via 5-HT2AR antagonism with 5-HT1AR partial agonism. Collectively, relatively low doses of lurasidone improved executive functions in a significant proportion of atypical antipsychotic-resistant schizophrenia via different pharmacological mechanisms with conventional atypical antipsychotics (possibly 5-HT7R antagonism).

This hypothesis is supported by preclinical behavioral findings (70). Social withdrawal, which is a core negative symptom of schizophrenia, can be modeled in the social interaction test using N-methyl-D-aspartate receptor (NMDA)/glutamate antagonists in rodents (70, 71). Acute administration of SB269970 (a 5-HT7R antagonist) ameliorated ketamine-induced social withdrawal, whereas sulpiride was ineffective (72). Interestingly, the co-administration of an inactive low dose of SB269970 displayed the prosocial effects of sulpiride (72). Another behavioral study demonstrated that lurasidone ameliorated the deficits of novel object recognition induced by phencyclidine and was antagonized by AS-19 (5-HT7R agonist) (22). Therefore, D2R antagonism with 5-HT7R antagonism may be a novel candidate pharmacological profile of an atypical antipsychotic class. In particular, the D2R, 5-HT1AR, 5-HT2AR, and 5-HT7R occupancies mediated by the approved dose of lurasidone (80 mg) should be determined by employing PET.

### Mood and Anxiety Disorders

A Bayesian network meta-analysis reported that lurasidone was more efficacious than aripiprazole and ziprasidone, and demonstrated comparable efficacy to quetiapine and olanzapine monotherapies for the management of bipolar depression (73). The efficacy of lurasidone in the acute treatment of bipolar depression, as both monotherapy and adjunctive therapy to lithium/valproate, has been reported in clinical trials (74, 75). Therefore, the general efficacy of lurasidone for the treatment of bipolar depression seems to be comparable with other mood-stabilizing atypical antipsychotics, whereas neither the superiority nor specific targets of the clinical efficacy of 5-HT7R antagonism have been demonstrated.

Although 5-HT7R variants are not associated with response to 5-HT7R antagonistic atypical antipsychotics in schizophrenia (see section Schizophrenia), a promoter single-nucleotide polymorphism in 5-HTR7 gene, rs7905446, which increases 5-HT7R expression (76), was positively associated with response to SSRI in individuals with mood disorders (76). These clinical findings indicates that 5-HT7R antagonism probably provides antidepressant-like action.

Major depression with mixed-features is regarded as conventional antidepressant-resistance and is associated with suicide ideation, manic switching agitation, and impulsivity; however, the standard medication for the mixed-features variant is yet to be clarified (77, 78). The specific “mixed-features variant” was incorporated in the Diagnostic Statistical Manual of Mental Disorders 5th edition. Therefore, the clinical efficacy of conventional mood-stabilizing atypical antipsychotics and antidepressants, except for lurasidone, remain to be clarified (78). A randomized double-blind, placebo-controlled study and its *post-hoc* studies demonstrated that lurasidone improved symptoms of depression/mania, anxiety, and irritability in post-menopausal women (79–82). Furthermore, a double-blind placebo control study revealed that lurasidone improved depressive symptoms without affecting manic symptoms in mixed-feature variants (83). Several studies have compared mood-stabilizing atypical antipsychotics (including olanzapine, lurasidone, brexpiprazole, ziprasidone, and cariprazine) with placebo, assessing the efficacy in the acute phase of presumptive mixed-feature variants (40, 84, 85). The available studies support the expected benefits of mixed-feature variants, whereas the overall effect may be similar in these mood-stabilizing atypical antipsychotics (40, 84, 85). To explore the efficacy of 5-HT7R antagonism against mixed-feature variants, the detailed clinical effects of conventional mood-stabilizing atypical antipsychotics and antidepressants on mixed-features variants need to be clinically investigated.

Preclinical findings using selective 5-HT7R antagonist and 5-HT7R knockout mice suggest that 5-HT7R inhibits both antidepressant-like and anxiolytic-like effects (21, 24, 86, 87). A selective 5-HT7R antagonist, SB269970, exhibited anti-immobility-like and antidepressant-like effects in the forced swim and tail suspension tests (21, 24). Furthermore, 5-HT7R knockout mice displayed tolerability to depression-like behavior in these tests (21, 88). These preclinical behavioral findings suggest that activation of 5-HT7R contributes to the pathomechanisms of depression. 5-HT7R antagonism mediated by SB269970 or JNJ18038683 produced antidepressant-like effects and promoted the antidepressant effects of several antidepressants, including citalopram, imipramine, desipramine, and moclobemide (24, 89, 90). Notably, SB269970 displayed rapid-acting antidepressant effects (91).

Based on the fast-acting antidepressant-like effects of 5-HT7R antagonists (91), several psychiatrists anticipated the development of a novel rapid-onset antidepressant class when compared with conventional antidepressants prescribed in clinical settings. Available medications using monoamine transporters that inhibit antidepressants and psycho-behavioral therapies require more than several weeks for the onset of beneficial effects (92). The delay of conventional monoaminergic antidepressants and psycho-behavioral therapies remains one of the major drawbacks of current treatments for depressive disorder, and faster-acting antidepressants are needed for patients with suicidal tendencies (93). Unfortunately, the onset of the antidepressant effect of lurasidone seems to require a duration equivalent to those of conventional antidepressants (73, 80, 82, 94, 95). However, a recent clinical trial demonstrated that both intravenous and oral administrations of vortioxetine displayed significant improvements in depression (Montgomery Åsberg Depression Rating Scale and Hospital Depression Scale) and anxiety (Hospital Anxiety Scale) after 3 days (95). Vortioxetine is a high-affinity inhibitor of human 5-HT transporter (Ki = 1.6 nM), 5-HT3R (Ki = 3.7 nM), 5-HT7R (Ki = 19 nM), and an agonist of 5-HT1AR (Ki = 15 nM) (Table 1) (36). Although the affinity of vortioxetine to rat 5-HT7R (Ki = 200 nM) is lower compared to human 5-HT7R (96), subacute administration (within 3 days) of effective dose of vortioxetine rapidly downregulates rat 5-HT7R (97). These preclinical demonstrations suggest that vortioxetine is a relatively low affinity to 5-HT7R compared to other 5-HT receptor subtypes, but suppresses 5-HT7R function with rapid 5-HT7R downregulation as an inverse agonist, similar to other 5-HT7R inhibiting mood-stabilizing atypical antipsychotics, clozapine, lurasidone, and olanzapine (97, 98). In other words, the rapid-acting antidepressant and anxiolytic actions of 5-HT7R antagonism have not been completely refuted and are worth reassessing after future clinical findings have accumulated.

Local hippocampal administration of SB269970 produced antidepressant-like activity in the rat forced swim test (87). Thus, blockade of 5-HT7R in the hippocampus might be beneficial in depression. Enhanced serotonergic transmission (activation of 5-HT1AR) plays an important role in the anti-depressive mechanisms of selective 5-HT reuptake inhibitors, but hippocampal 5-HT7R suppression is probably required for anti-depressive action. Acute stress increased 5-HT7R mRNA expression in the hippocampus (99). The anxiolytic-like effects of SB269970 were revealed in the Vogel drinking test, the elevated plus-maze test, and the four-plate test in mice (24). Local administration of SB269970 exhibited anxiety-like effects in the Vogel conflict test (87). SB269970 decreased the number of marbles buried in the marble-burying test (86). These findings suggest the effectiveness of 5-HT7R antagonists in the treatment of obsessive-compulsive disorder and anxiety disorders. 5-HT7R knockout mice exhibited similar behaviors in mice treated with SB269970 and antidepressants (21, 88); however, 5-HT7R knockout mice showed decreased immobility in forced swim and tail suspension tests and decreased both the duration and frequency in the rapid-eye-movement sleep phase (21, 88).

### Neurodevelopmental and Neurodegeneration Disorders

The majority of approved agents for the treatment of tic disorders and Tourette syndrome, aripiprazole, clozapine, olanzapine, quetiapine, risperidone, and pimozide, which reportedly reduce the severity of tic disorders and Tourette syndrome, are weak 5-HT7R antagonists (Table 1) (100–102). It is generally known that α2/α2A adrenoceptor agonism reduces the severity of tic disorders and Tourette syndrome (100, 102), whereas these agents are insensitive or α2 adrenoceptor antagonists (Table 1). Taken together with the *in vitro* binding profiles, 5-HT7R antagonism is a candidate target for the improvement of neurocognition.

Activation of 5-HT7R during adolescence induced persistent upregulation of 5-HT7R (17), and LP211 (selective 5-HT7R agonist) enhanced learning and memory in Fmr1 knockout mice, a genetic fragile-X syndrome mouse model (20). During adolescence, SB266970 enhanced impulsive behavior but attenuated the methylphenidate-induced reduction of impulsivity (103). These preclinical findings suggest the clinical potential of 5-HT7R agonists for the treatment of autism spectrum disorder and fragile-X syndrome (congenital X-linked disease associated with autistic traits and cognitive deficits) (20, 25). Conversely, chronic exposure to methylphenidate during postnatal and adolescence probably provides persistent structural rearrangements of the brain reward pathways associated with 5-HT7R (103). Therefore, the effects of 5-HT7R activation on neuronal plasticity during early development are not limited to embryonic and early postnatal development, but can also persist during adolescence and adulthood.

Although the 5-HT7R density decreased in correlation with age-dependent spatial memory deficits, which is possibly compensated by the 5-HT7R agonist (104), both 5-HT7R antagonists lurasidone and vortioxetine could clinically improve global cognitive performance (94, 105). Indeed, lurasidone is also significantly efficacious in older adults (≥55 years old) with bipolar depression (94), and bipolar depression comorbid with attention-deficit hyperactivity disorder (ADHD) in children and adolescents (106). The clinical improvement in cognitive disturbances in the elderly (94) and neurodevelopmental disorders (100, 102, 106) of 5-HT7R antagonism are in line with the preclinical findings on the effects of 5-HT7R antagonists on transmitter release. However, preclinical studies have previously reported more potentially important mechanisms associated with 5-HT7R antagonism. It is well-known that neurodegenerative processes and neurite retraction are considered to play important roles in the pathomechanisms of dementia and neurodevelopmental disorders (107). Preclinical findings support the clinical advantages of these 5-HT7R antagonistic agents owing to protection via inhibition of active polymerization and neurite retraction.

### Cognition

Both 5-HT1AR partial agonism and 5-HT7R antagonism improved executive functions in a mouse model, but 5-HT7R agonism failed to demonstrate this effect (108). Lurasidone improved executive function, but the selective 5-HT1AR antagonist, WAY100635, blocked the ability of lurasidone (108). These results indicate that the combination of 5-HT7R antagonism and 5-HT1AR partial agonism plays an important role in executive functioning. The cognitive promoting action of 5-HT7R antagonist is constructed by 5-HT1AR sensitive hippocampal-dependent and other hippocampal-independent neural circuits (25, 109, 110). Executive functions have been long known to involve the frontal cortex, and two projections from the hippocampus and thalamus (111). Mediodorsal thalamic nucleus (MDTN), which regulates outputs to the frontal cortex via integration of sensory and emotional inputs, is an essential partner of the frontal cortex in mediating executive functions (71, 112–115). Along with findings on 5-HT7R expression regions (14, 16, 18, 19, 25), the thalamocortical pathway is a candidate cognitive bottom-up regulation system (113, 115) associated with 5-HT7R antagonism.

Tonic hyperactivation of thalamocortical glutamatergic transmission has been observed in patients and experimental animal models of schizophrenia, ADHD, and autism (39, 112, 116–121). Although enhancement of serotonergic transmission plays an important role in the clinical efficacy of several atypical antipsychotics and conventional antidepressants, activation of serotonergic transmission to the MDTN, at least partially, negatively affects executive functions associated with the thalamocortical pathway via activation of excitatory postsynaptic 5-HT7R in the MDTN (18, 38, 122). A potent 5-HT1AR and 5-HT7R agonist, 8-hydroxy-2-(di-n-propylamino)tetralin (8-OH-DPAT) (9) suppresses several types of learning (25, 110). 8-OH-DPAT administration suppressed retention performance in passive avoidance training; however, SB269970 facilitated the negative effects of 8-OH-DPAT on the passive avoidance task for emotional learning (110). 5-HT1AR and 5-HT7R are expressed in hippocampal CA3 dendritic and neuronal cell body regions, respectively (123, 124). Administration of a selective 5-HT1AR antagonist and selective 5-HT7R agonist enhanced emotional memory in the passive avoidance behavior test (125). These findings indicate that 5-HT7R enhances emotional memory via hippocampal serotonergic transmission independent of 5-HT1AR.

Both aripiprazole and clozapine suppressed the tonic activation of thalamocortical glutamatergic transmission via activation of group II and III metabotropic glutamate receptors in the frontal cortex, respectively (116, 117). Contrary to aripiprazole and clozapine, lurasidone suppressed tonic hyperactivation of thalamocortical glutamatergic transmission through inhibition of postsynaptic 5-HT7R in the MDTN (18, 38). The different pharmacodynamic suppressive mechanisms of lurasidone and aripiprazole/clozapine on tonic hyperactivation of thalamocortical glutamatergic transmission suggest that the complementary effects of lurasidone on the rational integration of signaling inputs to the thalamocortical pathway regarding thalamic executive function are more effective than those of clozapine. Therefore, the cognitive (executive function) promoting effects of adjuvant lurasidone on atypical antipsychotic-resistant schizophrenia are probably demonstrated when tonic hyperactivation of thalamocortical glutamatergic transmission cannot be sufficiently improved by other atypical antipsychotics, via additional inhibition of excitatory 5-HT7R in the MDTN. The 5-HT7R antagonist facilitated the consolidation and reconsolidation of contextual fear conditioning memory (23). Local administration of SB269970 into the basolateral amygdala, but not the hippocampal CA1 region, facilitated the extinction of contextual fear conditioning memory (109). 5-HT7R inhibition appears to facilitate memory processes in broader cortico-limbic circuits, but not the hippocampus alone (109). Thus, 5-HT7R inhibition probably improves contextual learning and memory through a mechanism independent of the hippocampus (25). 5-HT7R may contribute to effective switching between hippocampus-dependent and hippocampus-independent learning strategies (126).

### Others

Recent findings have elucidated the role of 5-HT7R in a wide range of physiological functions in the mammalian CNS and peripheral organs (127–131). Noticeable modulation of peripheral 5-HT7R is expected to progress to the development of clinical applications in the medical field for autoimmune diseases and carcinoma. In peripheral tissues, the majority of 5-HT is produced by enterochromaffin cells of the gut mucosa and various immune cells. Peripheral 5-HT, which is produced in T lymphocytes and mast cells, is one immune system modulator that affects various immune cells via 5-HT receptors (3). A clinical study reported that several psychiatric disorders, including bipolar disorder, anxiety disorder, and major depression, are highly comorbid with inflammatory bowel diseases (around 50%) (132). Functional abnormalities of the serotonergic system contribute to the pathogenesis/pathophysiology of inflammatory bowel diseases (133). Silencing 5-HT7R, which is predominantly expressed in the immune system (134), reduced the severity of inflammation in an ulcerative colitis experimental mouse model (135). These findings suggest that functional abnormalities in 5-HT7R signaling in the brain, gut, and immune cells are involved in several inflammation- and immune-induced psychiatric disorders in the gut and brain.

A comprehensive *in vivo* screen test demonstrated that 297 psychoactive agents were 18-fold more likely to exert antiproliferative effects when compared with a random molecule population (136). Numerous clinical and meta-analysis studies reported that despite most patients with schizophrenia smoking heavily, the pooled overall cancer incidence rates for these patients were lower than their cancer risk factor exposure (136). Based on this clinical evidence, several antipsychotics are attracting attention as candidates for new treatment options for brain cancers owing to their ability to cross the blood-brain barrier (31). 5-HT7R antagonists have been proposed to block the growth of glioblastomas via inhibition of extracellular-regulated kinase (Erk) and interleukin 6 activities (137).

## 5-HT7R and Signal Transduction

### Effects of 5-HT7R on Organizing and Remodeling of Neural Circuits

**Figure 1:**
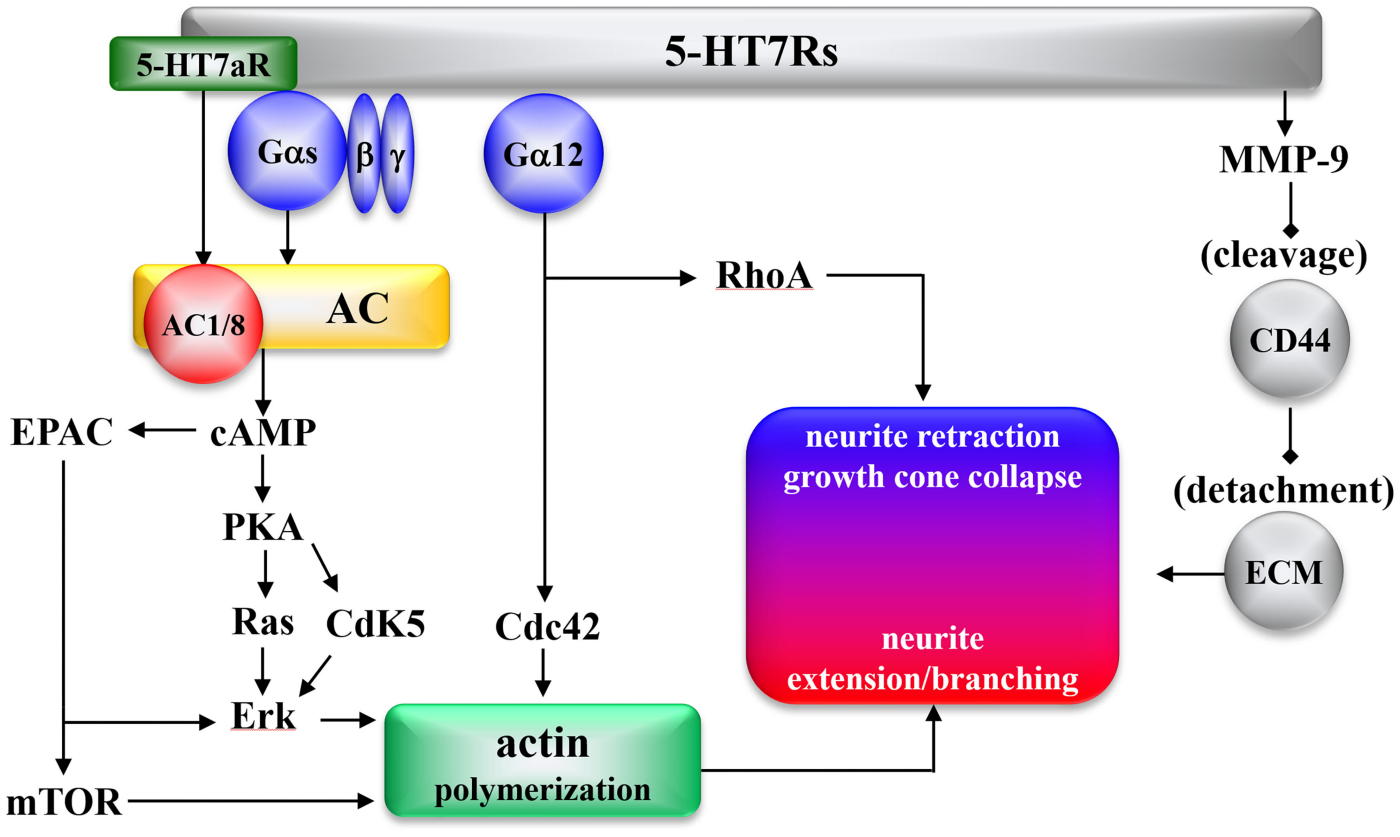
Morphogenic signaling mediated by the 5-HT7R. AC, Adenylyl cyclase; cAMP, cyclic adenosine monophosphate; Cdc42, cell division cycle protein 42; Cdk5, cyclin-dependent kinase 5; EPAC, exchange protein directly activated by cAMP; Erk, extracellular-regulated kinase; mTOR, mammalian target of rapamycin; PKA, protein kinase A; RhoA, Ras homolog gene family member A; CD44, hyaluronic acid receptor; MMP9, metallo proteinase 9; ECM, extracellular matrix.

The effects of 5-HT7R on intracellular signal transduction are summarized in Figure 1. 5-HT7R expresses several functional splice variants, distinct in their carboxyl terminals due to introns in the 5-HT7R gene (11, 138–140). Splice variants of 5-HT7R (5-HT7Ra, 5-HT7Rb, and 5-HT7Rc in rodents, and 5-HT7Ra, 5-HT7Rb, and 5-HT7d in humans) have been established (11, 98, 138–141). Functional differences among the splice variants include the 5-HT7Ra isoform activating types 1 and 8 of adenylyl cyclase (AC) Gs-independently (Figure 1) (142), and the internalization pattern of 5-HT7Rd differs from those presented by other isoforms (98, 141).

To clarify the mechanism of 5-HT7R on neuronal plasticity and cognitive and mood regulation, psychopharmacological studies have focused on the impact of 5-HT7R on neurodevelopmental processes, including migration, axon guidance, dendrite development, synapse formation, and nerve wiring during the early developmental stage.

5-HT7R was found to activate several signaling pathways involved in molecular mechanisms, such as Gαs and Gα12 proteins, which underlie neural remodeling (Figure 1). 5-HT7R activates AC via activation of Gαs activity (10). Apart from Gαs, the 5-HT7Ra isoform activates AC1 and AC8 via Ca^2+^/calmodulin-dependent and Gs-independent signaling (142). Increased cyclic adenosine monophosphate (cAMP) stimulates both protein kinase A (PKA) and subsequent activation of cyclin-dependent kinase 5 (Cdk5) (19, 143), Ras (144, 145), and exchange protein directly activated by cAMP (EPAC) (146), resulting in Erk signaling activation (144, 147). Furthermore, 5-HT7R-induced Ras and EPAC signaling promote the activation of mammalian target of rapamycin (mTOR) (Figure 1) (148).

Additionally, 5-HT7R activates several signaling pathways associated with Gα12 (143). 5-HT7R/Gα12 activates both Ras homolog gene family member A (RhoA) and cell division cycle protein 42 (Cdc42) (143, 149). Another 5-HT7R pathway was recently reported: in the detachment from the extracellular matrix (ECM), 5-HT7R cleaves the extracellular domain of the hyaluronic acid receptor (CD44) via activation of metalloproteinase-9 (MMP9) (150). The detachment from ECM via CD44/MMP9 plays an initial role in both dendritic spine remodeling and synaptic pruning, followed by neurite retraction by RhoA and neurite extension/branching by mTOR, Erk, and Cdc42 (Figure 1).

The serotonergic system, which is one of the initial organizing systems in development, generates neurogenesis, cell migration, axon guidance, dendritogenesis, synaptogenesis, and brain wiring throughout life (151). The impact of 5-HT7R on neuronal morphology in early developmental stages plays a fundamental role in the establishment and maintenance of neural connectivity and synaptic plasticity. During the embryonic stage, 5-HT7R induces neurite outgrowth of cortical, striatal, and hippocampal neurons via activation of Cdk5 and mTOR (145, 152). 5-HT7R is thought to contribute to the modulation of synaptic plasticity and neuronal connectivity during the developing and mature brain (128). Chronic 5-HT7R activation generates dendritic spine formation and increases the number of structurally intact synapses and expression of α-amino-3-hydroxy-5-methyl-4-isoxazolepropionic acid (AMPA)/glutamate receptor in hippocampal neurons (143). Neural circuits can be remodeled, induced by reactions to physiological and pathological inputs well into adulthood, continuing to exhibit robust plasticity throughout the entire lifespan of individuals (153). During the pre- and postnatal periods, exposure to selective serotonin reuptake inhibitors generates long-term anxiety in adulthood without affecting the morphological alterations of the brain (104, 154). Therefore, reorganization of dendritic morphology induced by 5-HT7R signaling provides new synapse growth and initial neuronal network formation, which is the target of event-related structural and functional plasticity in the early developmental stage (Figure 1) (155, 156).

A recent study suggest that rs300774, which is a candidate variant of LMWPTP associated with suicide, various transmission, including 5-HT and GABA (157). It has been reported that CD44 plays as a signaling receptor for LMWPTP induction (158), whereas redox-dependent downregulation of RhoA activity is modulated by oxidative modification of low-molecular weight protein tyrosine phosphatase (159). Therefore, the interaction between downstream signaling of LMWPTP and 5-HT7R possibly provides us a novel pathophysiological hypothesis regarding mood disorder and/or suicidal ideation. Furthermore, it has been demonstrated that a heterodimer and homodimers composed of 5-HT1AR and 5-HT7R, together with monomers, coexist in the cells (160). The heterodimer suppresses and enhances the stimulatory effects of 5-HT1AR on G-protein-gated inwardly rectifying potassium channels and mitogen-activated protein kinases, respectively. Interestingly, the heterodimer enhances the internalization of 5-HT1AR (160). Considering that the highest affinity for complex formation was obtained for the 5-HT7R/5-HT7R homodimers, followed by the 5-HT7R/5-HT1AR heterodimers and 5-HT1AR/5-HT1AR homodimers, determination of the effects of vortioxetine and lurasidone on the functional interactions between the heterodimer, homodimers, and monomers of 5-HT1AR and 5-HT7R could possibly clarify the complicated action of subchronic administration of these agents.

### Effects of 5-HT7R on Transmission Associated With Neuronal Plasticity

5-HT7R knockout mice displayed impaired long-term potentiation (LTP) in the hippocampus, and impairments in contextual learning, seeking behavior, and allocentric spatial memory (161, 162). Functionally, 5-HT7R activates neuronal excitability and LTP in the hippocampus of juvenile rodents without affecting those of adult individuals (163). Notably, 5-HT7R enhanced population spike amplitude and bursting frequency in the hippocampal CA1 and CA3 regions, respectively (164, 165). 5-HT7R-induced activation of cAMP/PKA signaling enhanced NMDA-evoked currents, resulting in the enhancement of the population spike amplitude and bursting frequency in hippocampal CA1 and CA3 regions, respectively (164–166). Furthermore, 5-HT7R activates hippocampal transmission postsynaptically owing to enhanced phosphorylation of the GluA1 AMPA (α-amino-3-hydroxy-5-methyl-4-isoxazolepropionic acid)/glutamate receptor, induced via cAMP/cAMP response element-binding protein (CREB) signaling (167, 168). Reportedly, 5-HT7R reverses long-term depression (LTD) associated with metabotropic glutamate receptors (20). These electrophysiological findings describe the molecular mechanisms underlying the positive effects of 5-HT7R on cognition and memory.

Activation of 5-HT7R during adolescence induces persistent upregulation of 5-HT7R (17). Chronic exposure to methylphenidate during postnatal and adolescence probably provides persistent structural rearrangements of the brain reward pathways associated with 5-HT7R (103). The molecular mechanism underlying 5-HT7R-induced remodeling increases neurite and dendritic spine elongation via matrix metalloproteinase (MMP)-9/CD44 and Cdc42 in reversal learning and neuronal regeneration (150). Therefore, the effects of 5-HT7R on neuronal plasticity during early development are not limited to embryonic and early postnatal development but can also persist in adolescence and adulthood.

Pathologically, activation of 5-HT7R during adolescence leads to increased dendritic arborization in the nucleus accumbens, which is one of the most important neural circuits associated with the pathophysiology of schizophrenia (169–171).

### Effects of 5-HT7R on Transmission After Maturation of the Nervous System

Microdialysis studies demonstrated that acute administration of a therapeutically relevant lurasidone dose increased the extracellular levels of dopamine, L-glutamate, and norepinephrine without affecting those of 5-HT or γ-aminobutyric acid (GABA) in the frontal cortex (Table 2) (18, 38, 172, 184). The effects of systemic administration of therapeutically relevant doses of conventional atypical antipsychotics on transmitter release are summarized in Table 2. The profile of lurasidone on transmitter release in the frontal cortex is similar to quetiapine but not to aripiprazole, blonanserin, clozapine, risperidone, or zotepine (18, 38, 116, 117, 172–181). D2R blockade with enhanced dopamine release in the frontal cortex produces the fundamental psychopharmacological effects of atypical antipsychotics (29). The combination of D2R inhibition with 5-HT2AR inhibition or 5-HT1AR activation contributes to enhanced dopamine release in the frontal cortex (185). Additionally, GABAergic disinhibition in the frontal cortex probably provides enhanced dopamine release induced by aripiprazole and clozapine (174, 176); however, lurasidone-induced dopamine release is not modulated by regional GABAergic disinhibition, similar to blonanserin, quetiapine, risperidone, and zotepine. These discrepancies in the frontal transmitter release profiles among 5-HT7R antagonistic antipsychotics (lurasidone, aripiprazole, clozapine, quetiapine, risperidone, and zotepine) suggest that 5-HT7R antagonism probably does not play an important role in enhanced dopamine release in the frontal cortex mediated by these antipsychotics. Therefore, 5-HT7R antagonism is possibly more effective in cognitive impairment and mood disturbances than against core (positive and negative) symptoms of schizophrenia.

**Table 2:** Summary of effects of the systemic administration of therapeutic-relevant dose of antipsychotics and 5-HT7R antagonist on transmitter release in the frontal cortex.

	**LUR**	**BNS**	**APZ**	**CLZ**	**QTP**	**RIS**	**ZTP**	**SB269970**
Norepinephrine	↑	↑	→	↑	↑	↑	↑	↑
Dopamine	↑	↑	↑	↑	↑	↑	↑	↑
5-HT	→	→	→	→	→	↑	→	↑
Glutamate	↑	→	→	↑	↑	→	↑	→
GABA	→	→	↓	↓	→	→	↑	
References	(18, 38, 172)	(172, 173)	(116, 174, 175)	(117, 175–177)	(178)	(173, 179, 180)	(181)	(182, 183)

*(2R)-1-[(3-Hydroxyphenyl)sulfonyl]-2-[2-(4-methyl-1-piperidinyl)ethyl]pyrrolidine (SB269970). ↑, increase; ↓, decrease; →, no change*.

Acute systemic administration of selective 5-HT7R antagonist, (2R)-1-[(3-hydroxyphenyl) sulfonyl]-2-[2-(4-methyl-1-piperidinyl)ethyl]pyrrolidine (SB269970) increased the basal release of norepinephrine and dopamine without affecting their metabolites in the medial prefrontal cortex (mPFC) (182). In contrast to catecholamines, acute systemic administration of SB269970 increased the basal release of 5-HT and 5-hydroxyindole acetic acid (5-HIAA) in the mPFC (183). Therefore, inhibition of 5-HT7R contributes to increased monoamine release in the mPFC; however, the mechanisms of catecholamine and 5-HT release induced by 5-HT7R inhibition vary, as catecholamine metabolites are not affected by 5-HT7R inhibition.

Serotonergic neurons in the DRN, which are regulated by inhibitory GABA_A_ receptor and 5-HT1AR, project into GABAergic interneurons in the DRN, glutamatergic neurons in the frontal cortex, and the MDTN (18, 38, 39, 186). GABAergic interneurons in the DRN are regulated by both excitatory 5-HT7R and NMDA/glutamate receptors (18, 38, 39, 186). In DRN slice patch-clamp investigations, inhibition of 5-HT7R generated depolarization and increased neuronal firing frequency via attenuation of spontaneous inhibitory postsynaptic potential (sIPSP) (183, 186). A multiprobe dialysis study demonstrated that under 5-HT1AR blockade, inhibition of 5-HT7R reduced GABA release; however, during 5-HT1AR activation, the stimulatory effects of SB269970 on 5-HT release in the DRN could not be observed (18, 39, 186, 187). Therefore, enhanced 5-HT release in the DRN activates 5-HT1AR and GABAergic inhibition, resulting in the suppression of serotonergic neurons in the DRN. Collectively, these findings indicate that inhibition of 5-HT7R suppresses GABAergic negative feedback in the DRN, increasing 5-HT release in the frontal cortex (186).

In contrast to 5-HT, local administration of SB269970 into the mPFC weakly but not significantly increased regional basal release of norepinephrine and dopamine (38). Although local administration of SB269970 into the frontal cortex has not been demonstrated to increase regional catecholamine release, the regulation system in the frontal cortex is probably not the region responsible for the increased basal region catecholamine release induced by systemic SB269970 administration. In particular, the regions responsible for catecholamine release induced by systemic SB269970 administration might occur outside the cortex (182).

Clinical and preclinical studies have emphasized that disturbance of the MDTN is particularly relevant for cognitive dysfunction characterized by developmental disorders and psychoses, including autism spectrum disorder, intellectual disability, ADHD, mood disorder, epileptic psychosis, and schizophrenia (112, 118, 119, 188–193). MDTN receives several types of inputs from the cortical and subcortical regions associated with learning, memory, emotion, and perceptual integration (194–196). MDTN-lesioned monkeys continued to respond to stimuli after being satiated with associated food rewards (191). Thalamocortical glutamatergic transmission is considered to play a role in maintaining flexible stimulus–reward associations (120, 122, 197). Therefore, enhancement of the sensitivity and reliability of MDTN signaling partially regulates the promotion processes of wide range cognitive functions via the integration of emotional and sensory information.

**Figure 2:**
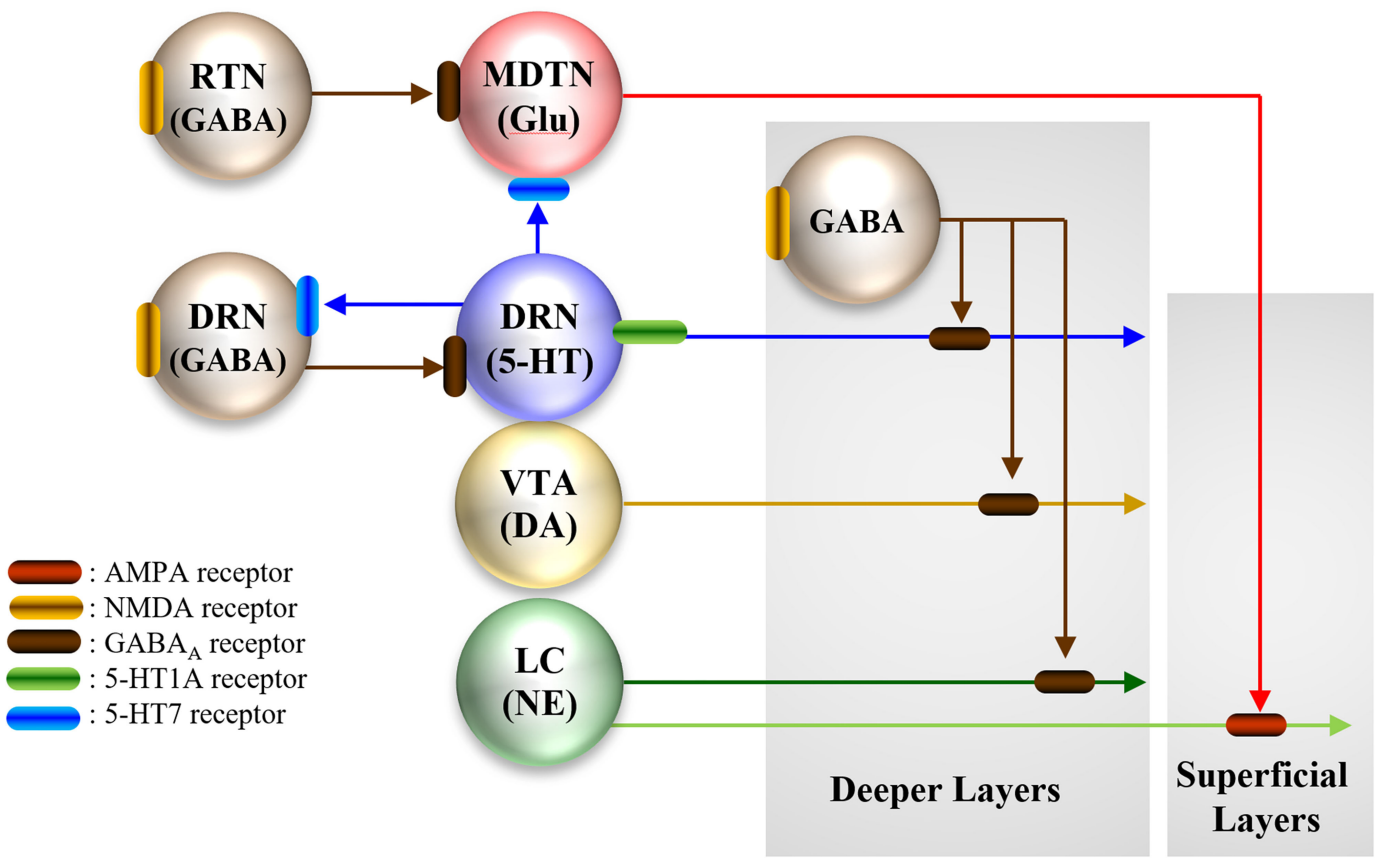
Proposed hypothesis for the extended complicated neural circuit connectivities involved in thalamocortical glutamatergic pathway, from the reticular thalamic nucleus (RTN), mediodorsal thalamic nucleus (MDTN) to the frontal cortex, mesothalamic serotonergic pathway, from the dorsal raphe nucleus (DRN) to the MDTN, mesocortical noradrenergic pathway, from the locus coeruleus (LC), dopaminergic pathway, from the ventral tegmental area (VTA), and serotonergic pathway from the DRN to the frontal cortex.

Monoaminergic neurons in the DRN, ventral tegmental area (VTA), and locus coeruleus (LC) project selective terminals to deeper layers of the frontal cortex, whereas part of the catecholaminergic neurons in the LC project co-releasing terminals (dopamine and norepinephrine) to superficial layers (Figure 2) (178, 181). The co-releasing terminals presynaptically receive excitatory AMPA/glutamate receptors from MDTN (Figure 2) (112, 120, 122, 178, 181, 186). Continuously hyperactivated MDTN glutamatergic transmission results in the relative deterioration of sensitivity to input signaling from other regions, similar to the functional disruption of MDTN activity (193). Recent preclinical studies demonstrated that several cognitive promoting agents, guanfacine, memantine, aripiprazole, and clozapine, compensate for the hyperactivation of thalamocortical glutamatergic transmission due to modulation of α2A adrenoceptor, system Xc-, and metabotropic glutamate receptors (116, 117, 120, 121). MDTN is regulated by excitatory serotonergic and inhibitory GABAergic inputs via the 5-HT7R and GABA_A_ receptors, respectively (39, 112, 116–121). Similarly, lurasidone compensated for hyperactivation of thalamocortical glutamatergic transmission via inhibition of 5-HT7R (18, 38). The mechanisms of 5-HT7R inhibition on complicated interactions between thalamocortical glutamatergic and mesocortical catecholaminergic pathways in the frontal cortex provided the first glimpses of the pathophysiology underlying hippocampal-independent cognitive-modulatory functions associated with 5-HT7R (Figure 2) (25, 126).

## Remaining Challenges and Conclusion

Despite the advantages of 5-HT7R antagonism, the following challenges need to be resolved to develop effective clinical applications of 5-HT7R modulation for the treatment of numerous disorders involving cognitive impairments.

1) Although 5-HT7R antagonism possibly improves cognitive impairments, the 5-HT7R expression in the frontal cortex of patients with schizophrenia is lower than that in healthy controls (129, 130). Subchronic administration of the 5-HT7R agonist (LP211) and antagonists (SB269970) upregulated and downregulated 5-HT7R expression, respectively (17, 98). Therefore, reduced 5-HT7R expression in patients with schizophrenia cannot be determined to be directly involved in the pathogenesis of schizophrenia; these findings might have been induced by 5-HT7R antagonism of several antipsychotics. In particular, the inhibition of 5-HT7R is a candidate for the treatment of schizophrenia.

2) Although sleep disturbance (reduced duration and frequency of rapid-eye-movement sleep phase) was observed in 5-HT7R knockout mice (21), a clinical study failed to detect any sleep disturbance (sleep onset, rapid-eye-movement, or slow-wave sleep) in individuals receiving lurasidone (198). Additionally, another clinical study reported that lurasidone did not affect the sleep phase, whereas 5-HT7R knockout mice exhibited sleep phase disruption (21). Along with 5-HT7R downregulation induced by long-term administration of lurasidone, sleep disturbance caused by lurasidone remains one of the side effects necessitating attention.

3) Cognitive impairment in the elderly positively is known to correlate with an age-dependent reduction of 5-HT7R expression (104); however, both vortioxetine and lurasidone can improve cognitive decline and in the elderly (94, 105). Preclinical findings support the clinical advantages of these 5-HT7R antagonistic agents owing to their inhibitory action on active polymerization and neurite retraction. We speculated the clinical advantage of 5-HT7R antagonism in elderly individuals; however, long-term exposure to 5-HT7R antagonists could facilitate the fragility of transmission associated with 5-HT7R in elderly individuals.

4) Reportedly, approved agents for the treatment of behavioral manifestations of autism spectrum disorder, including pimozide, aripiprazole, and risperidone, exhibit a 5-HT7R antagonistic profile (28); however, the effectiveness of 5-HT7R agonists for the treatment of autism spectrum disorder and fragile-X syndrome was demonstrated in experimental models (20, 25). These discrepancies suggest the fundamental pathomechanisms of neurodevelopmental disorders, as these have been considered a dysfunction of both development and/or collapse of the neural circuit system. Neurodevelopmental dysfunction can be compensated by 5-HT7R-induced enhancement of the remodeling of neuronal connectivity (possibly collapse and branching). In contrast, after the maturation of neural circuits, the disruption of neuronal connectivity can be prevented by 5-HT7R inhibition. In particular, a possible crucial therapeutic time-window exists based on the pathomechanism of each disorder for the 5-HT7R modulating agent. In this review, we postulate that 5-HT7R inhibition might contribute to the stability of neuronal connectivity and transmission; however, rational enhancement of remodeling through 5-HT7R modulation may provide us with a novel strategy for various other neuropsychiatric disorders.

5) In this review, we hypothesized that the clinical efficacy of the approved lurasidone dose (80 mg) is mediated predominantly via D2R and 5-HT7R antagonism, rather than 5-HT2AR antagonism with 5-HT1AR partial agonism according to the PET and receptor binding studies. It is unlikely that our assumptions will be surpassed as the positive relationship between receptor occupancy and the binding affinity of most antipsychotics has been well-demonstrated. The 5-HT7R occupancy level of the approved lurasidone dosage should be clarified.

## Author Contributions

MO: conceptualization, writing—original draft preparation, project administration, and funding acquisition. RO, TH, and KF: validation. RO, TS, and MO: writing—review and editing. All authors contributed to the article and approved the submitted version.

## Conflict of Interest

The authors declare that the research was conducted in the absence of any commercial or financial relationships that could be construed as a potential conflict of interest.
